# Catalytic Esterification of Levulinic Acid into the Biofuel n-Butyl Levulinate over Nanosized TiO_2_ Particles

**DOI:** 10.3390/nano12213870

**Published:** 2022-11-02

**Authors:** Shuolin Zhou, Lu Wu, Junzhuo Bai, Min Lei, Min Long, Keying Huang

**Affiliations:** School of Elementary Education, Changsha Normal University, Changsha 410100, China

**Keywords:** TiO_2_ nanoparticles, solid acid catalyst, n-butyl levulinate, esterification, response surface methodology

## Abstract

Levulinic esters, synthesized by the esterification of biomass-derived levulinic acid with various alcohols, is an important chemical that plays an essential role in the fields of biomass fuel additives, organic synthesis, and high value-added products. In the present work, the catalytic esterification of levulinic acid with n-butyl alcohol was selected as a typical model reaction to investigate the catalytic performance of an inexpensive commercial catalyst, titanium oxide nanoparticles. The influences of reaction time, reaction temperature, and catalyst loading on the conversion of levulinic acid to n-butyl levulinate were systematically examined through single-factor experiments. Additionally, the optimization of the reaction conditions was further investigated by a Box–Behnken design in response to the surface methodology. The desired product, n-butyl levulinate, with a good yield (77.6%) was achieved under the optimal conditions (reaction time of 8 h, reaction temperature of 120 °C, and catalyst dosage of 8.6 wt.%) when using titanium oxide nanoparticles as catalysts. Furthermore, it was found that addition of water to the catalytic system facilitated the reaction process, to some extent. This study reveals that the nanosized TiO_2_ material_,_ as an efficient solid acid catalyst, had good catalytic performance and stability for the esterification of levulinic acid after six consecutive uses.

## 1. Introduction

The contradiction between the continuous consumption of fossil fuels and the rapid growth of energy demand has become more and more intense, motivating people to explore and develop renewable energy sources for achieving a sustainable future [1,2]. Biomass is regarded as the only resource of organic carbon that can be regenerated by capturing carbon dioxide, and it is potentially the most sustainable renewable energy [3,4,5] and has recently received a great deal of attention. It is therefore of great significance to explore and develop an efficient production strategy for biomass-based platform chemicals, which will effectively relieve the energy shortage and expand the fine chemical industry chain [6,7].

Levulinic acid (LA) is considered one of the most important biomass-derived platform molecules which can currently be efficiently obtained from biomass on a large-scale [8,9,10]. Extensive and continuous efforts have been devoted to the use of LA to offer many other bio-based products with widespread applications, such as fuel additives, high-value chemicals, and biopolymers [11,12,13,14,15]. Among these LA derivatives, levulinic esters (LEs) are regarded as a highlighted fuel additive or as a hydrocarbon fuel due to their high lubricity and high stability. In addition, LEs can be further applied to an important precursor for producing a variety of high-value chemicals, including γ-valerolactone [16,17,18,19,20,21,22], pyrrolidone [23], 1,4-pentanediol [24], succinic acid [25], etc. Among multiple reaction routes, the direct catalytic conversion of LA to LEs is an efficient and simple pathway in terms of a high atom economy and a low separation cost, and it has gained remarkable attention [9,10]. Under the advocacy of green chemistry, the replacement of homogeneous catalysts by solid catalysts has gradually become a current research trend. Hence, the development of green and environmentally friendly, benign heterogeneous catalysts for the synthesis of LEs from LA would be highly desirable and required [26,27]. Recently, various heterogeneous catalysts including zirconium phosphate [28], sulfonated mesoporous carbon [29], methane sulfonic acid [30], ion-exchange resins [31], sulfonic acid-functionalized materials [32,33,34], heteropolyacids [35,36,37], and biocatalysts [38,39] have been employed to catalytic LA to produce LEs. However, some heterogeneous catalysts often suffer from the complex catalyst preparation process, the severe leaching of catalytic active species, and an unstable catalyst structure during the esterification reaction process, which limits their use in practical applications.

The use of nanoparticles as heterogeneous nano-catalysts has attracted considerable attention, and it has been widely studied. Among the various nano-catalysts, titanium dioxide (TiO_2_) is considered a promising nanomaterial in heterogeneous catalysis due to its fascinating and interesting physical and chemical properties. Recently, nanostructured TiO_2_ has been intensively used as a solid acidic catalyst in organic reactions such as the aldol condensation of furfural [40], synthesis of 5-(hydroxymethyl) furfural [41,42], esterification of free fatty acids with ethanol [43], and alcoholysis preparation of diosgenin [44]. Moreover, TiO_2_ nanomaterials are promising candidates for green organic synthesis owing to their excellent properties, such as chemical stability, low cost, nontoxicity, and high reusability [45,46,47].

Herein, the catalytic performances of three commercial titanium dioxide nanomaterials for the transformation of biomass-derived LA into LEs were assessed and compared. The physicochemical properties of these nanosized TiO_2_ samples were characterized by XRD, TEM, NH_3_-TPD, and the Fourier transformation infrared spectroscopy (FT-IR) of pyridine adsorption techniques. The influence of reaction parameter such as time, temperature, and catalyst loading on the esterification of LA was studied and discussed in detail using a single factor method. Response surface methodology (RSM) based on a Box–Behnken design (BBD) was employed to optimize the experimental design and elucidate the interactions between the factors on the LA conversion. A fitted model with the desired product yield as the response value was established. In addition, the reusability of the nanosized TiO_2_ was also discussed.

## 2. Experimental

### 2.1. Materials and Reagents

Three commercial TiO_2_ nanoparticles in pure anatase phase with average particle sizes ranging from 5 to 60 nm (denoted as TNPs-1, TNPs-2, and TNPs-3) and levulinic acid (99.8%) were purchased from Aladdin Reagent (Shanghai, China) and used as received. The n-butyl alcohol and ethanol were obtained from Sinopharm Chemical Reagent Co., Ltd. (Shanghai, China) and used without further purification.

### 2.2. Characerization

X-ray diffraction (XRD) patterns of the TiO_2_ samples were recorded using a Bruker D8 Advance diffractometer with a Cu Kα radiation wavelength (λ = 1.5418 Å) at a scanning rate of 5°/min with the diffraction angle (2θ) ranging from 10° to 80°. Transmission scanning microscopy (TEM) was used to observe the morphologies and particle sizes of the TiO_2_ nanoparticles on an FEI Tecnai F20 electron microscope (American). The ammonia temperature-programmed desorption (TPD-NH_3_) was obtained on a BEL-Cat II apparatus (Microtrac BELCat II, Japan). The TiO_2_ samples (0.1 g) were placed in a tube and pretreated at 350 °C for 1 h with a flow of 30–50 mL/min helium, then cooled to 100 °C. A stream of 10% NH_3_/He was introduced until the samples were ammonia-saturated. After purging under helium for 1 h at 100 °C to remove the weak physical absorption of NH_3_ on the surface, the ammonia was desorbed using a 10 °C/min heating rate to 800 °C in a He atmosphere. The relative quantification of the acid sites was determined based on the curve of the ammonia desorption. The surface acidic property (Brønsted and/or Lewis acid) of the TiO_2_ samples was measured with pyridine FT-IR on a Bruker Tensor 27 FT-IR spectrometer (Germany). The TiO_2_ samples were heated at 200 °C for 1 h under vacuum, then exposed to purified pyridine vapors at 25 °C for 30 min. The physic-sorbed pyridine on the samples’ surfaces was degassed at 100, 200, and 300 °C under high vacuum, respectively.

### 2.3. Catalytic Performance Test

The catalytic activity of the nanosized TiO_2_ for LA esterification was tested in a round bottom flask equipped with a reflux condenser. In a typical experiment procedure, levulinic acid (3 mmol), n-butyl alcohol (30 mmol), and 10 wt.% of TiO_2_ nanoparticles (based on the LA mass) were added and then conducted at various temperatures ranging from 90–120 °C at atmospheric pressure, with vigorous stirring in an oil-washing oven. The reaction mixture was periodically withdrawn with a syringe, filtered, and analyzed using gas chromatograph (GC, Beijing Zhongke Huifeng Co., Ltd., Beijing, China). The GC was equipped with a fame ionization detector (FID) and an HP-5 capillary column. The temperatures of the injection port, the oven, and the detector were 250 °C, 150 °C, and 250 °C, respectively. Gas chromatograph-mass spectrometry (GC–MS, Shimadzu, Kyoto, Japan) was used to confirm the reaction intermediates.

### 2.4. Design of Experiment Using BBD

The BBD design was employed to systemically study the influence of mainly the operating parameters, including reaction time, reaction temperature, and catalyst loading, and the interactions of such variables on the conversion of the LA. Thus, a three factor, three-level BBD with a total of 15 runs was employed, and the experiments were conducted in a random manner to decrease errors. The results were analyzed using Design-Expert software (trial version 8.0.6.1, Stat-Ease, Inc., Minneapolis, MN, USA).

### 2.5. Catalyst Reusability Tests

The reusability of the TiO_2_ samples was tested under the optimal reaction conditions. In the LA esterification reaction, the TiO_2_ samples were easily recovered by centrifugation at 3000 rpm for 5 min, washed repeatedly with ethanol (3 × 5 mL), and dried at 60 °C overnight before a new run. The recovery rate of the TiO_2_ samples was determined based on the mass of TiO_2_ before and after the reaction.

## 3. Results and Discussion

### 3.1. Characterization of the TiO_2_ Nanoparticles

**Figure 1:**
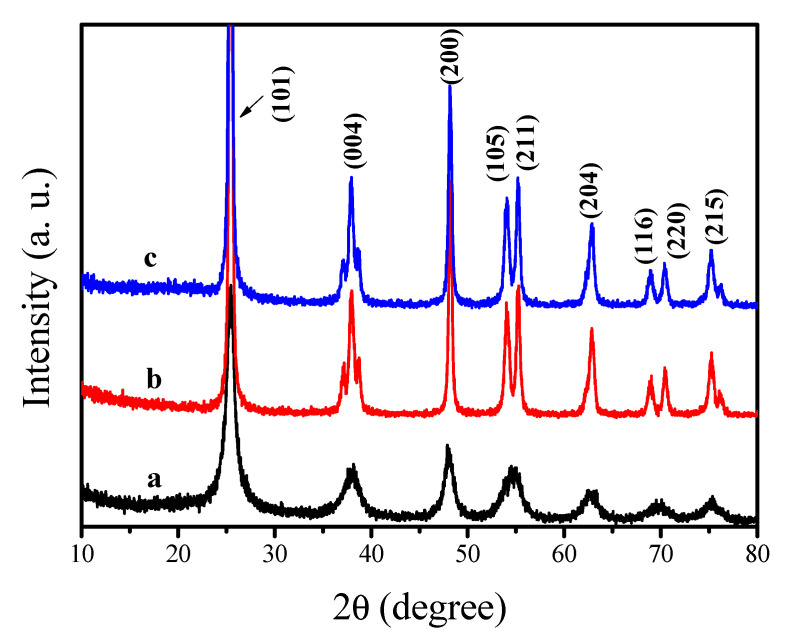
XRD patterns of (**a**) TNPs-1, (**b**) TNPs-2, and (**c**) TNPs-3.

Figure 1 compares the XRD patterns of the different TiO_2_ nanoparticles. The main peaks are at 25.6, 38.1, 48.0, 54.2, and 62.9 and belong to the (101), (004), (200), (105), and (204) planes of anatase for the three samples, confirming that all TiO_2_ nanoparticles were pure anatase phase. Compared with TNPs-1, the diffraction peaks of TNPs-2 and TNPs-3 increased in intensity and became sharper. It should be noted that all samples show the broad diffraction reflections, suggesting the possible existence of crystalline particles of a nano-scale size (<100 nm). The estimated crystal size was further calculated by the Scherrer equation. It is noteworthy that the average crystalline size of the TNPs-1 was estimated to be approximately 6.8 nm.

**Figure 2:**
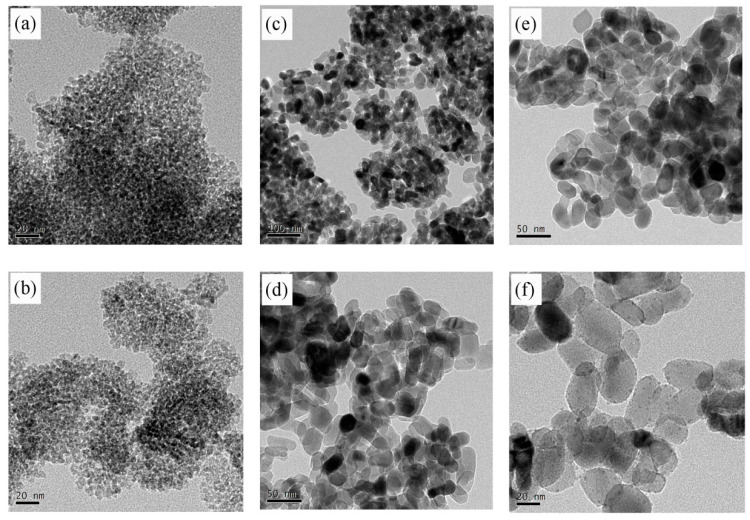
TEM graphs of the TNPs samples: (**a**,**b**) TNPs-1, (**c**,**d**) TNPs-2, and (**e**,**f**) TNPs-3.

The size of the primary TiO_2_ particles was further observed form the TEM images (Figure 2). The TEM images show that the TNPs-1 was dispersed and the TNPs-1 particles were relatively regular in shape. The magnified image shows that the TNPs-1 was entirely tetragonal, with a size of 3–7 nm. The particle size distribution of the TNPs-1 was consistent with the results of the XRD analysis. However, the TNPs-2 and TNPs-3 particles were not spherical but irregular, with a prolate or oblate shape. The particle sizes of TNPs-2 and TNPs-3 were in the range of 30–40 nm and 35–45 nm, respectively.

**Figure 3:**
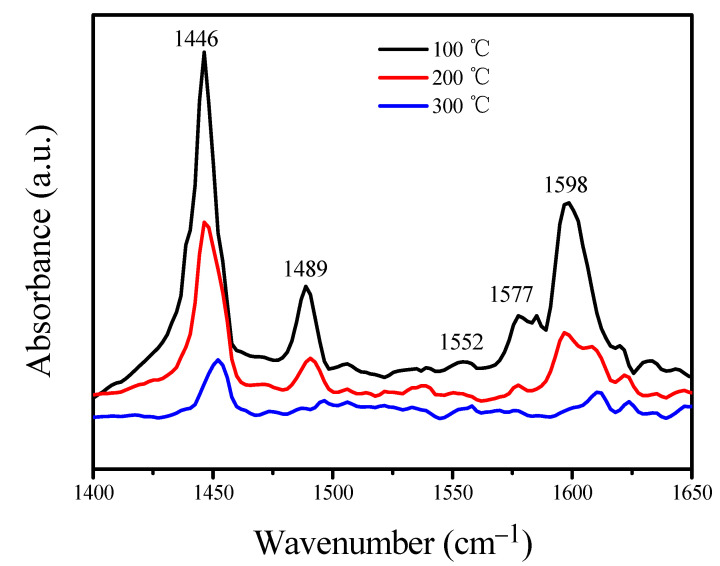
FTIR spectra of the pyridine adsorbed on TNPs-1.

Pyridine adsorption experiments on the TNPs were carried out to identify the presence of Lewis and/or Brønsted surface acid sites, and the results are displayed in Figure 3. There are two main characteristic peaks observed in the regions of 1400–1452 cm^−1^ and 1570–1620 cm^−1^. These absorption bands assigned to the pyridine molecules align with Lewis acid sites, suggesting the presence of Lewis acid sites in the TNPs. There is a weak characteristic peak observed at approximately 1555 cm^−1^, which is assigned to the adsorption of pyridine at the Brønsted acid sites [48]. In addition, the distinct band at approximately 1489 cm^−1^ is ascribed to both Brønsted and Lewis acid sites [49,50]. Moreover, it was seen that with an increase in the desorption temperature from 100 to 300 °C, these bands showed slow decreases in intensity. The above observations indicate that the strong Lewis acid sites were dominant in the TNPs. It is noted that the Lewis acid sites could convert to Brønsted acid sites in the presence of water, thus improving the reaction reactivity, to a certain degree.

**Figure 4:**
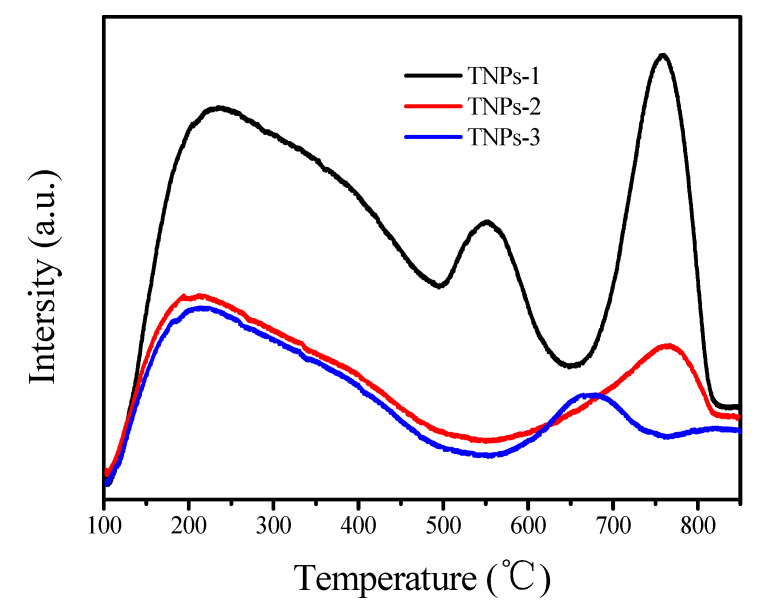
The NH_3_-TPD profiles of TNPs-1, TNPs-2, and TNPs-3.

Figure 4 shows the NH_3_-TPD spectra of TNPs-1, TNPs-2, and TNPs-3. As seen from Figure 4, there is a broad peak observed at approximately 210–230 °C for all samples. In addition, in the case of TNPs-1, two desorption peaks at higher temperatures of approximately 550 and 750 °C suggest a stronger acid strength [51]. However, a desorption peak at high temperatures for TNPs-2 (760 °C) and TNPs-3 (670 °C) was lower than that of TNPs-1, indicating a lower strong acid strength. The total acidity of TNPs-1, TNPs-2, and TNPs-3 was 1.005 mmol/g, 0.409 mmol/g, and 0.368 mmol/g, respectively.

### 3.2. Catalyst Performance of the TNPs

**Scheme 1:**
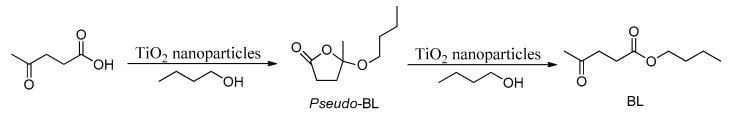
TiO_2_ nanoparticles catalyzed by the LA esterification into BL.

The catalytic performances of the different TNPs in the LA esterification reaction were investigated, and the results are summarized in Table 1. The LA conversion rate without any catalyst was low. Notably, the conversion of LA was 71.6%, 48.4%, and 43.5% when the TNPs-1, TNPs-2, and TNPs-3 were used as catalysts, respectively. The high catalytic activity of TNPs-1 may be because it has more acidic sites compared to TNPs-2 and TNPs-3. The catalytic performance of TNPs-3 was comparable to that of TNPs-2 because the total acid amount and particle size of both are close to each other. The catalytic performance of the commercial SiO_2_ nanoparticles was poor due to the lower concentration of acid sites. These results imply that the number of acid sites on the catalyst directly affects the catalytic activity. Moreover, two products, BL and *p*seudo-BL, were detected by GC-MS in the esterification reaction of LA with n-butanol. Similar phenomena for the reaction of intermediate *p*seudo-BL have been reported in several studies [52,53,54], and the reaction process can be described as shown in Scheme 1. Among these TNPs materials, TNPs-1 showed a good catalytic performance for LA esterification and was therefore selected for the experiments that followed.

### 3.3. Effect of Reaction Time on LA Esterification

**Figure 5:**
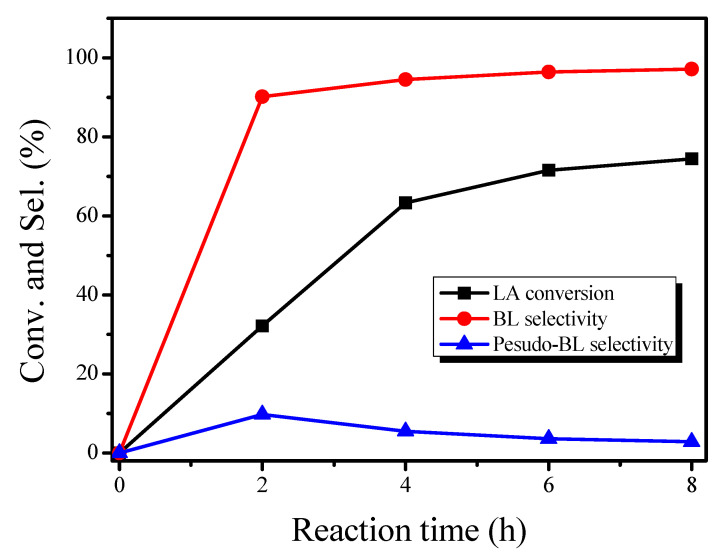
Influence of reaction time on the esterification of LA. Reaction conditions: LA: n-butanol = 1:10, catalyst loading: 10 wt.% (based on the LA mass), and reaction temperature: 120 °C.

In order to elaborate in detail the overall catalytic esterification process, the influence of reaction time on the conversion of LA was firstly evaluated using TNPs-1 as catalyst, and the corresponding results are depicted in Figure 5. It was observed that the LA conversion rate was increased with a prolonged reaction time, whereas the selectivity of *p*seudo-BL decreased. The selectivity of *p*seudo-BL kept a low level (approximately 5.5%) after 4 h of reaction. The selectivity of BL was 96.4% with a 71.6% conversion of LA after the reaction proceeded for 6 h. The above-described results suggest that the reaction of LA with n-butyl alcohol would firstly tend to generate *p*seudo-BL. It was noteworthy that the LA conversion had no remarkable change with further prolonging the reaction time to 8 h. This may be the reason that the esterification reaction reached a chemical equilibrium status, thereby presenting a slight increase in LA conversion and BL selectivity [55]

### 3.4. Effect of the Reaction Temperature on LA Esterification

**Figure 6:**
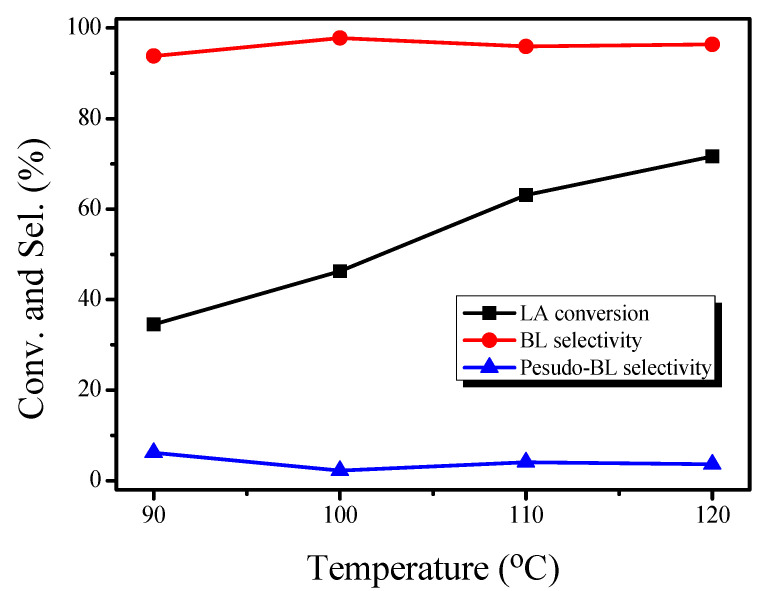
Influence of the reaction temperature on the esterification of LA. Reaction conditions: LA: n-butanol = 1:10, catalyst loading: 10 wt.% (based on the LA mass), and reaction time: 6 h.

The catalytic activities of the TiO_2_ nanoparticles were further tested at various reaction temperatures (90–120 °C) under other fixed reaction conditions: a reaction time of 6 h and TNPs-1 catalyst loading of 10 wt.%. Figure 6 illustrates the conversion of LA enhanced linearly with increasing the reaction temperature from 90 to 120 °C. Thus, the conversion of LA reached the maximum value of 71.6% at 120 °C. At the same time, it was observed that the selectivity of n-butyl levulinate was close to 100% in all tests. High temperatures were expected to promote the reaction equilibrium shift towards n-butyl levulinate, thereby improving the BL yield. These results clearly demonstrate that the reaction temperature has a great positive influence on the LA esterification.

### 3.5. Effect of the Catalyst Dosage on LA Esterification

**Figure 7:**
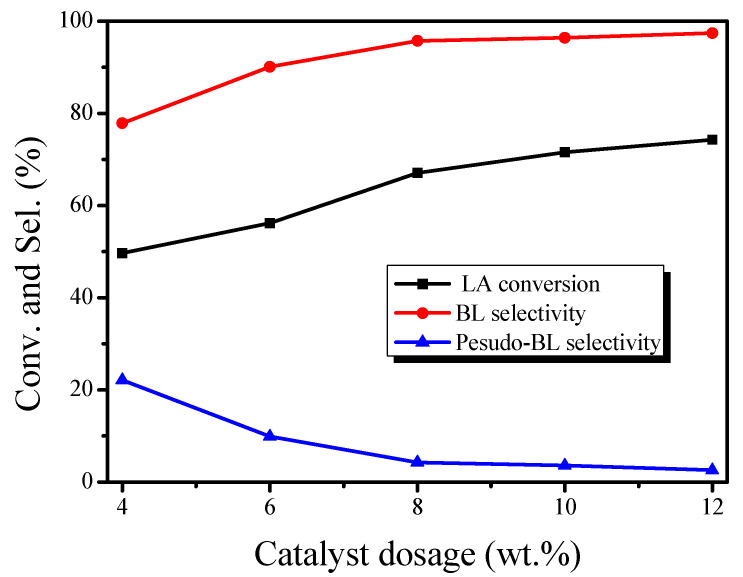
Influence of the catalyst dosage on the esterification of LA. Reaction conditions: LA: n-butanol = 1:10, reaction time: 6 h, and reaction temperature: 120 °C.

The influence of the TNPs dosage on the LA esterification was evaluated by the change in the mass of TNPs-1 in the range of 4 to 12 wt.% (based on the LA mass) and keeping all other parameters constant. It was clearly observed, as seen in Figure 7, that a low LA conversion with a relatively high selectivity of *p*seudo-BL was obtained when 4 wt.% of the catalyst dosage was added. As depicted in Figure 7, the increase in the LA conversion aligned with the increased catalyst dosage, which should be attributed to the increase in the number of availability active sites. When the 10 wt.% catalyst was added, the conversion of LA to BL showed the maximum conversion (71.6%), with a selectivity of 96.4% toward BL. However, the conversion of LA did not improve significantly with the further increase in catalyst dosage. This can be explained by the reaction reaching an equilibrium state and the excessive acidic sites were not necessary, resulting in a marginal increase in LA conversion. Furthermore, a high dosage of catalyst will limit the mass transfer rate, leading to lower catalytic activity [34,56].

### 3.6. BBD Design and Data Analysis

The BBD design and experiments were performed to optimize the reaction conditions, with full consideration of the interaction of the multi-factors. In this study, three variables and the corresponding level, catalyst dosage (A, 5–10 wt.%), reaction time (B, 4–8 h), and reaction temperature (C, 100–120 °C) were mainly considered and investigated. In this study, 15 set of experiments were conducted, and the center point experiment was repeated three times to decrease errors.

The experimental design matrix and the corresponding results are recorded in Table 2. A polynomial model regression for the three parameters was given and expressed as shown in Equation (1).

Y(%) = 60.20 + 7.69A + 5.71B + 10.10C − 1.22AB + 0.30AC + 2.75BC − 7.71A^2^ + 0.94B^2^ − 2.79C^2^, (1)

where Y is the yield of BL and A, B, and C are the catalyst dosage, reaction time, and reaction temperature, respectively.

**Figure 8:**
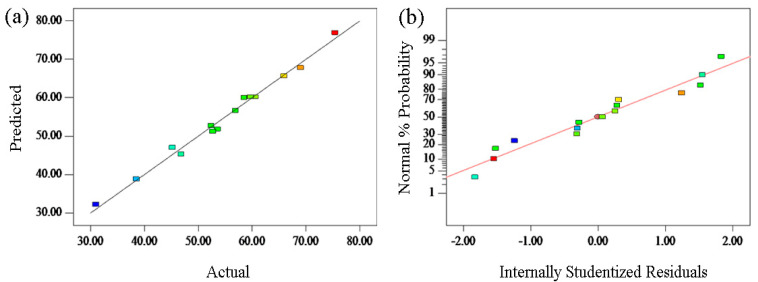
(**a**) Plots of the predicted vs. experimental results for the BL yield and (**b**) the normal probability plot of the residuals for the BBD model.

The experimental values of the BL yield versus the corresponding predicted values, as displayed in Figure 8a, show good agreement with a high R^2^ value (0.9897). This result demonstrates the validity of the model. Moreover, as revealed in Figure 8b, it was observed that the normal distribution of the residuals in the plot of residuals versus the predicted response was perfect due to these points being close to a straight line. The fitted model was further analyzed by analysis of variance (ANOVA), and the results are given in Table 3. If the *p*-value was lower than 0.05, this would indicate that the model’s terms were more statistically significant [57,58]. Based on this point, the F-value and the corresponding *p*-value for the model were found to be 53.41 and 0.0002, respectively, indicating that the fitted model is significant and can be successfully used to predict the LA esterification process. Furthermore, the “Adequate precision” value was 28.023 greater than 4, implying that the fitted model can describe the good relationship between the BL yield and the three variables [59].

The independent variables affect the catalytic activity in the following order: temperature (C) > catalyst dosage (A) > reaction time (B). This means that the reaction temperature was the major parameter affecting the esterification process, which is consistent with the above results. Furthermore, the interaction terms of the reaction time × temperature (BC) and the quadratic terms, such as catalyst dosage × catalyst dosage (A^2^) and temperature × temperature (C^2^), were highly significant to the BL yield because these *p*-values were as low as <0.05 (Table 3). Further, the interaction effects between any two independent variables on the yield of BL were evaluated. It was observed that the interaction between time (B) and temperature (C) (BC) on BL yield was significant (*p*-value = 0.0373). The three-dimensional (3D) response surface plots and contour plots were further represented in order to investigate the interaction effects between temperature and time on the yield of BL.

**Figure 9:**
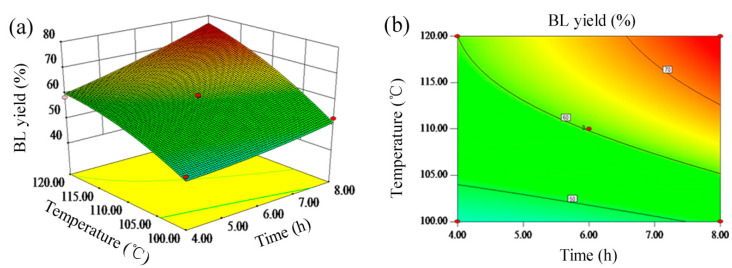
(**a**) 3D response surface plot and (**b**) contour plots for the mutual influences of reaction time and temperature on the BL yield.

Figure 9 shows that the yield of BL did not increase significantly with the reaction time at lower reaction temperatures. Further, it was noticeable that the BL yield remained at a low level at lower reaction times. The yield of BL showed an increase with the increase in reaction temperature from 100 to 120 °C at a relatively long reaction time (5–8 h). Notably, a rise in BL yield was observed, while the reaction temperature increased at longer reaction time levels. The predicted optimal esterification conditions, based on the obtained RSM model, were found to be a time of reaction of 8 h, a dosage of catalyst of 8.6 wt.%, and a temperature of the reaction of 120 °C. Under these reaction conditions, the model predicted that the experiment result of the BL was 78.4% and 77.6%, respectively. There is a negligible difference between the model’s predicted value and the experimental result, which indicates that the optimum conditions for LA esterification are reliable and accurate.

### 3.7. Reusability and Stability of the TNPs

**Figure 10:**
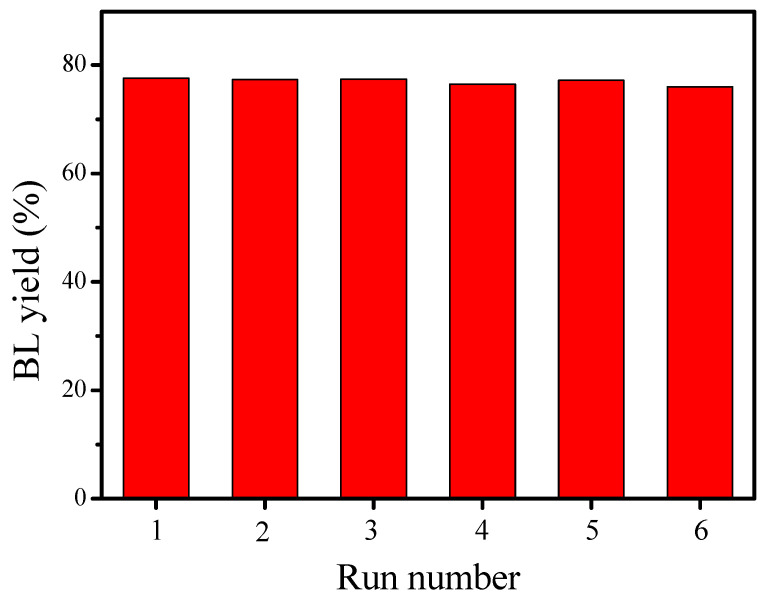
The reusability results of the TiO_2_ NPs.

**Figure 11:**
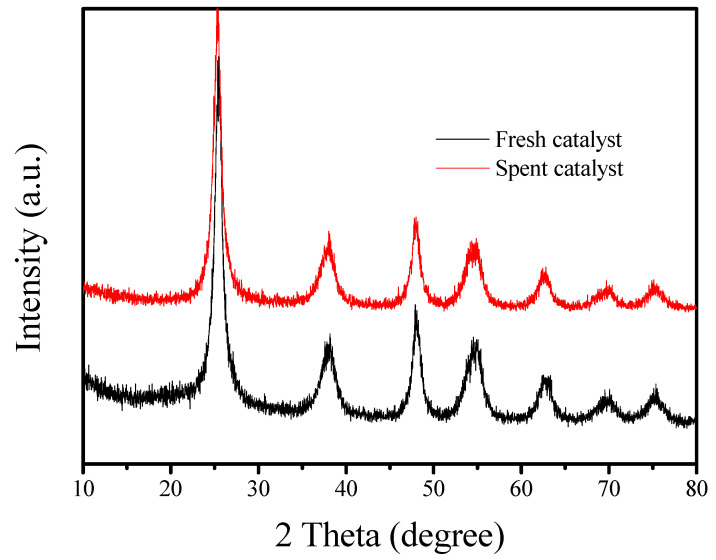
XRD pattern of the spent TNPs catalyst.

The stability of the TNPs-1 in the LA esterification reaction under the above optimal conditions was examined by performing consecutive batch reactions. After the reaction, TNPs-1 could be separated easily by centrifugation, and the recovery rate of TNPs-1 from the reaction mixture was quite high (>95%). The reusability results of the TiO_2_ nanoparticles are summarized in Figure 10. A small progressive decrease in BL yield was observed after six consecutive uses. This phenomenon may be attributed to a combination of reasons, such as a loss in mass of the catalyst and the adsorption of organic matter on the catalyst surface. Therefore, it can be inferred that the TNPs can be reused without a considerable loss in catalytic activity. Moreover, the spent catalyst was further characterized by XRD. As displayed in Figure 11, it was clearly shown that was no remarkable difference in the XRD pattern of the spent catalyst, indicating the TiO_2_ nanoparticles are stable for LA esterification.

In addition, the catalytic performances of the TiO_2_ nanoparticles were further compared with the reported catalysts. Amberlyst-15, which as a typical solid acid catalyst, gave a low yield of BL (55%). The CMK-8-SO_3_H catalyst only showed moderate catalytic activity towards LA esterification at high temperatures. Using NER@3DOM/m-OS biocatalyst facilitates the LA conversion into BL at low temperatures, achieving 74.6% of the BL yield. Other heterogeneous catalysts, such as WO_x_/mesoporous-ZrO_2_ and commercial H-Beta, are found to be efficient catalysts for the conversion of LA. It can be clearly seen from Table 4 that the pure TiO_2_ nanoparticles exhibited good catalytic performances for synthesis of n-butyl levulinate from LA.

### 3.8. Possible Reaction Mechanism

**Scheme 2:**
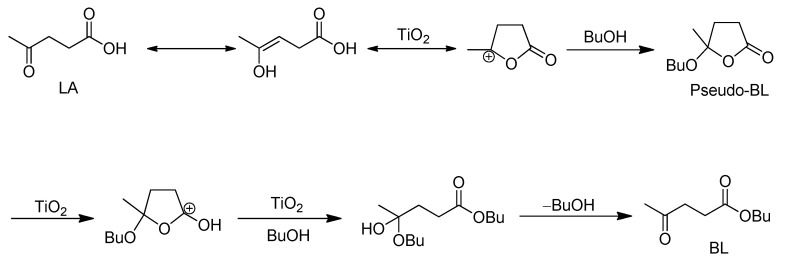
Proposed possible mechanism for generating BL from LA.

The reaction mechanism suggested for generating BL from LA is depicted in Scheme 2. An LA molecule was firstly adsorbed on the surface’s acidic sites, giving a protonated intermediate. Then, the presence of alcohol facilitated the corresponding intermediate towards *p*seudo-BL under the mild reaction conditions. Subsequently, the *p*seudo-BL underwent nucleophilic addition and ring opening to provide the final BL. A remarkable trans-esterification of the *p*seudo-BL into BL was clearly observed with the increased reaction time at the initial reaction period in the previous experiments (Figure 5). Moreover, the numbers of acid sites and the moderate acid strength in case of the TiO_2_ NPs are important factors for converting *p*seudo-BL to BL. Interestingly, we found that the yield of BL can be improved from 77.6% to 82.2% and 87.5% when 5 wt.% and 10 wt.% (based on the mass of the LA) water is added under the optimal conditions, respectively. The above results elucidate that the presence of the moderate water was expected to convert the surface Lewis acid sites into Brønsted acid sites, thereby enhancing the reactivity in the catalytic system. This will be very important for acid-catalyzed LA esterification in practical applications.

## 4. Conclusions

In conclusion, the esterification reaction of biomass-derived LA with *n*-butanol under solvent-free conditions using commercial TiO_2_ nanoparticles as the solid acid catalyst was investigated. The experimental results showed that a relatively high conversion of LA and a high selectivity of BL were obtained under mild conditions. The reaction model based on the BBD in RSM was built, and it was statistically significant. The reaction temperature was the major parameter affecting the esterification process. The optimum operation conditions were a catalyst dosage of 8.6 wt.%, a reaction temperature of 120 °C, and a reaction time of 8 h. It is highly important that the TiO_2_ catalyst could be easily separated for reuse, and it continued to exhibit a good catalytic performance after six repetition cycles. Moreover, the experimental results suggest that the conversion of LA to BL via *p*seudo-BL provides a more detailed description and better understanding of the reaction process. This work demonstrates that the nano-sized TiO_2_ materials are simple, green, and efficient candidate catalysts which have great potential for applications in biomass conversion.

## Figures and Tables

**Table 1:** Comparison of the various nanoparticles’ activities for LA esterification.

Entry	Sample	LA Conv. (%)	BL Sel. (%)
1	-	37.9	74.7
2	TNPs-1	71.6	96.4
3	TNPs-2	48.4	84.5
4	TNPs-3	43.5	95.8
5	SiO_2_ (commercial)	39.5	79.2

Reaction conditions: LA: n-butanol = 1:10, reaction temperature: 120 °C, reaction time: 6 h, and catalyst dosage: 10 wt.% (based on the mass of LA).

**Table 2:** Results of the BBD for the LA esterification optimizing process.

Run No.	Catalyst Dosage (A)	Reaction Time (B)	Temperature (C)	Y_exp_ (%)
1	5	4	110	38.5
2	10	4	110	56.9
3	5	8	110	52.4
4	10	8	110	65.9
5	5	6	100	31.0
6	10	6	100	45.2
7	5	6	120	53.6
8	10	6	120	69.0
9	7.5	4	100	46.8
10	7.5	8	100	52.7
11	7.5	4	120	58.5
12	7.5	8	120	75.4
13	7.5	6	110	60.3
14	7.5	6	110	60.6
15	7.5	6	110	59.7

**Table 3:** ANVOA for the optimized RSM model.

Source	Sum of Squares	DF	Mean Square	F-Value	*p*-Value Prob > F
Model	1834.68	9	203.85	53.41	0.0002
A-catalyst dosage	472.78	1	472.78	123.68	0.0001
B-reaction time	261.06	1	261.06	68.40	0.0004
C-temperature	816.08	1	816.08	213.83	<0.0001
AB	6.00	1	6.00	1.57	0.2652
AC	0.36	1	0.36	0.094	0.7711
BC	30.25	1	30.25	7.93	0.0373
A^2^	219.63	1	219.63	57.55	0.0006
B^2^	3.25	1	3.25	0.85	0.3988
C^2^	28.69	1	28.69	7.52	0.0407
Residual	19.08	5	3.82		
Pure error	0.42	2	0.21		
R^2^	0.9897				
Adj R^2^	0.9712				
C.V.%	3.55				
Adequate precision	28.023				

**Table 4:** Comparison of the catalytic performances for LA esterification using various catalysts.

Catalyst	Mole Ratio (LA:Alcohol)	Temp. (°C)	Time (h)	BL Yield (%)	Ref.
Amberlyst-15	1:20	117.7	5	55	[60]
CMK-8-SO_3_H	1:10	130	10	75.1	[29]
NER@3DOM/m-OS	1:10	40	12	74.6	[61]
WO_x_/mesoporous-ZrO_2_	1:5	120	2	64.9	[52]
Commercial H-Beta	1:7	120	4	82.2	[62]
TiO_2_	1:10	120	8	77.6	This work

## Data Availability

All data generated or analyzed during this study are included in this manuscript.
